# Deep Eutectic Solvent-Based Aqueous Two-Phase Systems and Their Application in Partitioning of Phenol Compounds

**DOI:** 10.3390/molecules29184383

**Published:** 2024-09-15

**Authors:** Isabela N. Souza, Lucas C. V. Rodrigues, Cleide M. F. Soares, Filipe S. Buarque, Ranyere L. Souza, Álvaro S. Lima

**Affiliations:** 1 Post-Graduated Program on Process Engineering, Tiradentes University, Aracaju 49032-490, SE, Brazil; isabela__souzza@hotmail.com (I.N.S.); cleide18@yahoo.com.br (C.M.F.S.); ranyerels@hotmail.com (R.L.S.); 2 Post-Graduate Program on Chemical Engineering, Federal University of Bahia, Salvador 40210-910, BA, Brazil; lucas.rodrigues@yahoo.com.br; 3 Instituto de Tecnologia e Pesquisa, Aracaju 49032-490, SE, Brazil; 4 Biochemical Engineering Department, School of Chemistry, Federal University of Rio de Janeiro, Rio de Janeiro 21941-909, RJ, Brazil

**Keywords:** deep eutectic solvents, aqueous two-phase system, partition, phenol compounds

## Abstract

This work studies the partition of phenolic compounds, namely caffeic acid, syringic acid, vanillic acid, ferulic acid, and vanillin, in aqueous two-phase systems (ATPSs) formed by acetonitrile and deep eutectic solvents (DESs) based on choline chloride ([Ch]Cl) and carbohydrates (sucrose, d-glucose, d-mannose, arabinose, and d-xylose). The binodal curves built at 25 °C and 0.1 MPa using DES were compared with ATPS composed of [Ch]Cl and the same carbohydrates. The ability to form ATPS depends on the number and kind of hydroxyl groups in DES’s hydrogen-bond donor compound (carbohydrates). ATPS based on DES showed biphasic regions larger than the systems based on [Ch]Cl and carbohydrates alone due to the larger hydrophilicity of DES. The ATPS were used to study the partition of the phenolic compounds. For all the systems, the biomolecules preferentially partitioned to the acetonitrile-rich phase (K > 1), and the best recovery in the top phase ranged between 53.36% (caffeic acid) and 90.09% (vanillin). According to the remarkable results, DES-based ATPS can selectively separate ferulic acid and vanillin for the top phase and syringic, caffeic, and vanillic acids for the bottom phase, achieving a selectivity higher than two.

## 1. Introduction

Phenolic compounds are secondary metabolites of plants with one or more aromatic rings, which allows their classification into some sub-classes, such as phenolic acids, flavonoids, stilbenes, and lignans [1]. Phenolic acids, with one carboxylic group, present two distinct carbon frameworks: the hydroxycinnamic (ferulic and caffeic acids) and hydroxybenzoic (vanillic and syringic acids) structures [2]. Although the basic skeleton remains, they differ in the number and position of the hydroxyl groups on the aromatic ring [3]. The health-promoting benefits of phenolic compounds have attracted industrial interest due to their biological and pharmacological properties, namely antioxidant [4], anti-inflammatory [5], and anticarcinogenic activities [6], among others. They are also used in the food industry as antioxidants, improving color stability and sensory acceptance [7]. Daily, humans consume phenolic acids, which are in the estimated range of 25 mg to 1 g depending on the diet [8].

These compounds can be extracted from natural matrixes using processes such as solvent extraction [9,10], pressurized fluid extraction [11,12], supercritical fluid extraction [13,14], ultrasound-assisted extraction [15,16], and microwave-assisted extraction [17,18]. In addition, after extraction, purification protocols such as aqueous two-phase systems have been applied to obtain the target molecule with high purity levels [19,20].

Aqueous two-phase systems (ATPSs) are systems that have been successfully used in the extraction, concentration, and purification of different biocompounds such as enzymes [21], anthocyanins [22], flavonoids [23], dye [24], antibodies [25,26], and phenolic compounds [27,28] among others due to their versatility, biocompatibility, high efficiency, high yield, selectivity, low cost, ease scale-up, and technological simplicity [29]. ATPS are formed when two soluble compounds are mixed above a critical concentration, creating two immiscible phases [30].

A wide range of compounds has been used as ATPS constituents. Initially, they were based on polymer–polymer mixtures [31]. However, the viscosity and high cost led to the development of new systems constituted of polymer salt [32]. Since 2003, those ATPS formed of ionic liquid salt [33,34], ionic liquid carbohydrates [35], and ionic liquid polymers [36] have been used. Recently, the efforts of the literature have been focused on the use of alcohol salt [37], tetrahydrofuran carbohydrates [38], ethanol ionic liquids [39], and the application of DES as a constituent of ATPS [40,41].

Organic solvents appeared as a low-cost alternative to form ATPS with low viscosity that could be easily separated from the target biomolecule [42]. Acetonitrile (ACN) is a colorless, aprotic, and water-soluble solvent used to prepare ATPS [43]. Industrially, ACN plays an important role in pharmaceutical, cosmetic, and agrochemical processes [44]. DESs are a novel set of solvents prepared by mixing two or more cheap and safe compounds, which can interact with each other through hydrogen bonds and van der Walls forces to form a eutectic mixture [45,46,47]. These interactions between the hydrogen-bond donor (HBD) and hydrogen-bond acceptor (HBA) are the driving force for reducing the melting point of DES when compared with individual components [48]. Usually, quaternary ammonium salts are used as HBA (choline chloride, fitic acid, proline, and betaine), while metallic salts, alcohols, and sugars are employed as HBD [49,50,51]. Thus, the solvents have advantages such as a wide range of possible combinations, water compatibility, low vapor pressure, non-flammability, biocompatibility, and biodegradability [52].

Therefore, the main focus of this study was to determine binodal curves based on DES (formed by choline chloride and carbohydrates) + acetonitrile + water as an environmentally friendly and low-cost method for partitioning phenol compounds.

## 2. Results and Discussion

### 2.1. Binodal Curves

The binodal curves are shown in molality units. This representation was used to remove any effects related to the differences in the molecular weight of sugar studied to interpret the mechanisms responsible at the molecular level for phase separation. In all the phase diagrams, the two-phase region is above the solubility curve, while the single-phase region is below. The diagrams displaying a larger area above the binodal curve (immiscibility region) have a greater ability to form two phases.

**Figure 1:**
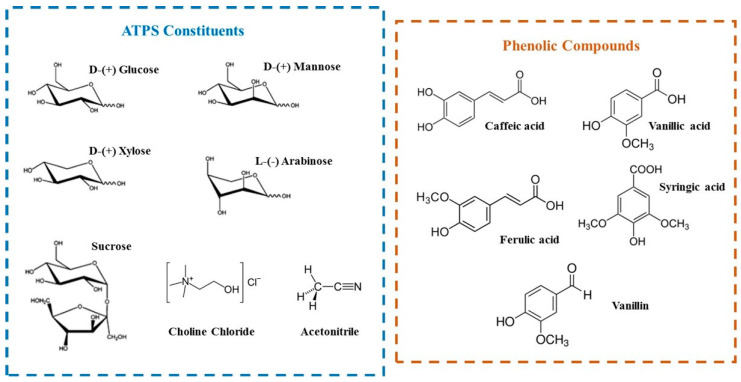
Chemical structure of ATPS constituents and phenolic compounds used in this work.

The ability to form ATPS composed of ACN + [Ch]Cl or ACN + carbohydrates (d-glucose, d-mannose, arabinose, d-xylose, and sucrose) has been reported in the literature by [53,54]. Moreover, it is known that these compounds ([Ch]Cl and carbohydrates) can form DES [52,55], and some DESs have been used in the preparation of ATPS with salts [40], polymers [56], and ACN [57]. However, no previous report on the formation of ATPS based on DES formed by [Ch]Cl + carbohydrates and ACN has been reported. Figure 1 depicts the chemical structure of the compounds used to form DES, then ATPS, and finally, the phenolic compounds partitioned.

**Figure 2:**
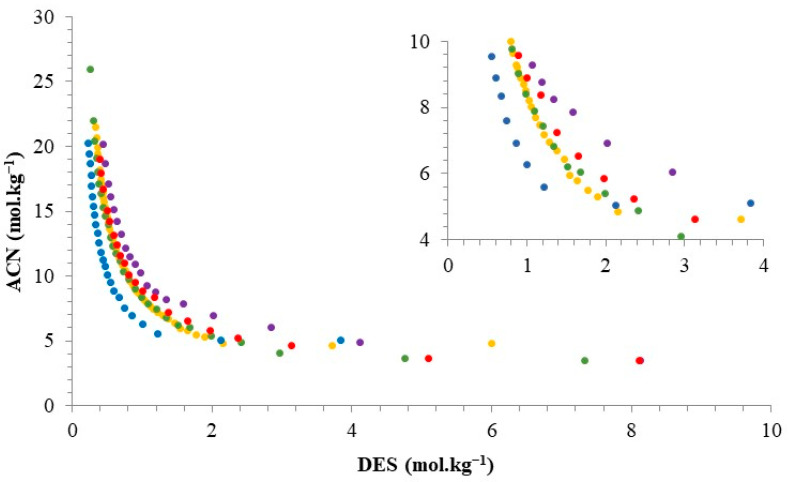
Binodal curves of ATPS based on ACN + DES (molar ratio 1:1) + water at 25 °C and 0.1 MPa. DES: (●) [Ch]Cl:Suc, (●) [Ch]Cl:Glc, (●) [Ch]Cl:Ara, (●) [Ch]Cl:Man, and (●) [Ch]Cl:Xyl.

In this work, we started by evaluating the effect of the DES hydrogen-bond donor on their phase formation capability. Figure 2 (Table S1 of Supplementary Materials) depicts the binodal curve of ATPS based on ACN + DES (molar ratio—1:1) + water at 25 °C and 0.1 MPa. In all the ATPS reported in this work, the top phase is rich in ACN, while the bottom phase is rich in DES.

The ability of DES to form two-phase systems is expressed by the proximity of the binodal curve to the origin of the Cartesian axis. The rank of biphasic system formation using DES ([Ch]Cl:Suc > [Ch]Cl:Glc ≈ [Ch]Cl:Man > [Ch]Cl:Ara > [Ch]Cl:Xyl) follows the same order previously observed for ATPS based on carbohydrates, and ionic liquids [34], ACN [54], or tetrahydrofuran [58]. The DESs employed in this study differ only on the carbohydrate used as a hydrogen-bond donor. The number of hydroxyl groups present in the disaccharide (sucrose–Suc) is higher than in monosaccharides (d-glucose—Glc, arabinose—Ara, d-mannose–Man, and d-xylose–Xyl), allowing a higher affinity and ability to form hydrogen bonds between these compounds and water, and consequently, to promote more intensely the sugaring-out effect and separation of phases [34,54]. The aldo-hexoses (with five hydroxyl groups), such as Gluc and Man, are more able to form hydrogen bonds and induce phase separation than aldo-pentoses (with four hydroxyl groups), namely Ara and Xyl [58]. The difference in the capability to phase separate for DES based on monosaccharides with an equal number of hydroxyls is related to the number of the more-accessible equatorial hydroxyls (Ne-OH): Glu (4.6), Xyl (3.5), Man (3.3), and Ara (2.6) [59,60]. The Ne-OH has the highest hydration capability, stabilizing the water structure [61,62].

**Figure 3:**
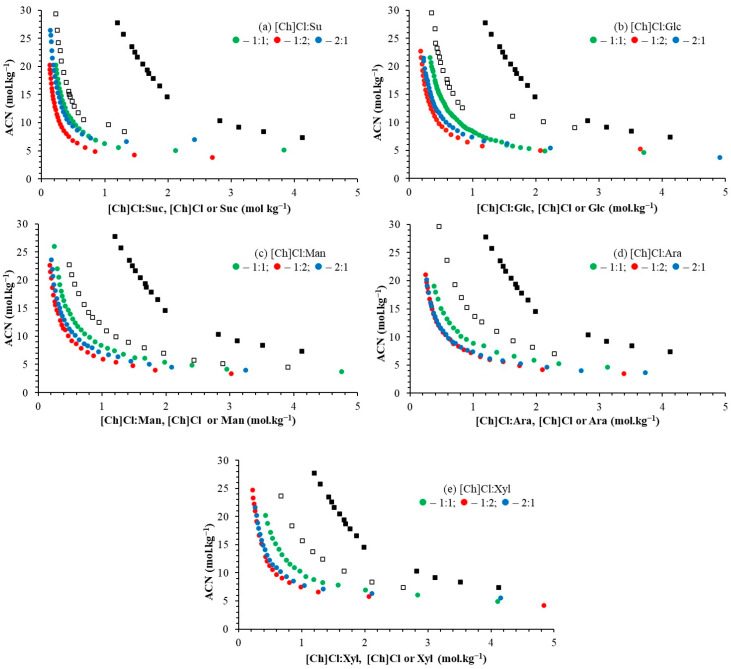
Binodal curves of ATPS based on ACN at 25 °C and 0.1 MPa. ACN + [Ch]Cl—■ [53]; ACN + carbohydrates—□ [54]; (**a**): [Ch]Cl:Su—(●—1:1; ●—1:2, and ●—2:1); (**b**): [Ch]Cl:Glc (●—1:1; ●—1:2, and ●—2:1); (**c**): [Ch]Cl:Man (●—1:1; ●—1:2, and ●—2:1); (**d**) [Ch]Cl:Ara (●—1:1; ●—1:2, and ●—2:1); (**e**) [Ch]Cl:Xyl (●—1:1; ●—1:2, and ●—2:1).

The effect of the molar ratio between the HBA:HBD ([Ch]Cl:carbohydrate) compounds of DES preparation was evaluated in the ATPS formation and is reported in Figure 3 and Tables S2 and S3 of Supplementary Materials), which can also compare the binodal curves based on ACN + DES with those formed by ACN + carbohydrates and ACN + [Ch]Cl [54].

In the systems formed here by DES + ACN + water, the order of ease of phase formation is as follows: 1:1 < 2:1 < 1:2 (HBA:HBD—[Ch]Cl:carbohydrate). In these systems, the DES constituents are more hydrophilic (expressed as log k_ow_—[Ch]Cl = −4.66; Suc = −4.53; Glc = −2.93; Man = −2.93; Ara = −2.94 and Xyl = −2.30) than acetonitrile (log k_ow_ = −0.17) [63]. Therefore, the higher the presence of the hydrophilic component, the easier the phase separation. Additionally, the formation of DES must provide more hydrophilicity to the constituent than pure constituents. For this reason, the systems formed by [Ch]Cl or carbohydrates are further away from the axes ([Ch]Cl < carbohydrates < DES) and consequently, less easily promote phase separation. Farias et al. [56], using aqueous two-phase systems formed by DES ([Ch]Cl:carbohydrates) and K_2_HPO_4_, observed an order opposite to that found in this work. This inversion is justified, as ACN is more hydrophobic than K_2_HPO_4_, requiring a more hydrophobic constituent than salt.

### 2.2. Partition of Phenolic Compounds

**Figure 4:**
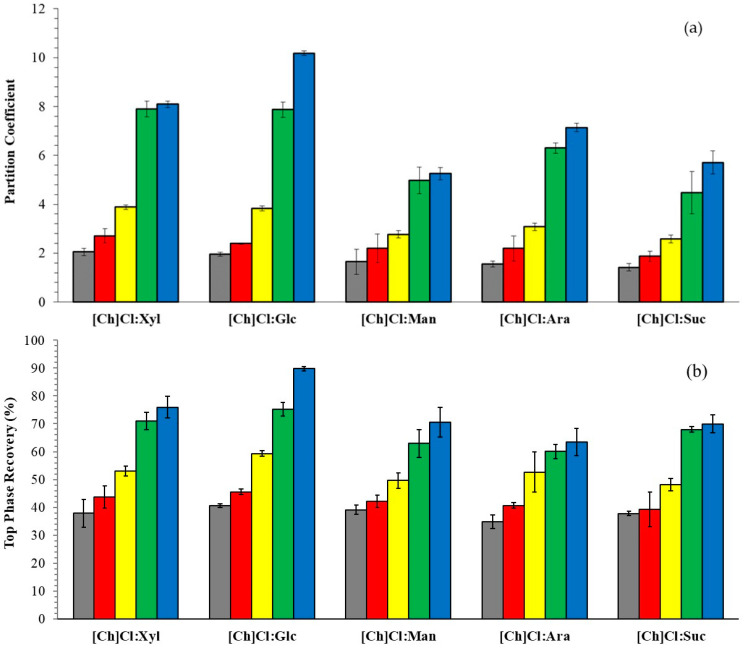
Partition coefficient (**a**) and recovery in top phase (**b**) for ATPS based on ACN + DES (molar ratio 1:1) + water at 25 °C and 0.1 MPa. ■—caffeic acid, ■—syringic acid, ■—vanillic acid, ■—ferulic acid, and ■—vanillin.

The application of the ATPS studied was investigated through the partitioning of theoretical phenolic compounds from their standards, such as caffeic acid (log k_ow_ = 1.424), ferulic acid (log k_ow_ = 1.671), vanillic acid (log k_ow_ = 1.324), syringic acid (log k_ow_ = 1.129), and vanillin (log k_ow_ = 1.19) [64]. Figure 4 (Tables S4 and S5 of Supplementary Materials) showed the partition coefficient (K) and recovery in the top phase (R_T_).

For the all the system, a 1:1 molar ratio was chosen, this would allow us to better understand the effects of partitioning without any preponderance of one of the DES constituents. The phenolic compounds partition to the ACN-rich phase (K >> 1) due to the hydrophobic nature of the biomolecules, which are more compatible with ACN (log k_ow_ = −0.17) than carbohydrates (−4.53 < log k < −2.30) and [Ch]Cl (log k_ow_ = −4.66) used to prepare the DES. Cardoso et al. [54] attributed the preferential migration of vanillin to the top phase to the hydrophobic characteristics of vanillin being compatible with acetonitrile, as well as reporting values close to the recovery of vanillin in the systems composed of ACN + carbohydrates. Le et al. [65] also demonstrated the preferential partition of phenolic compounds from coffee pulps to the ethanolic phase in the systems formed by ethanol, water, and (NH_4_)_2_SO_4_. In DES systems, the difference resided only in the carbohydrates (HBD), so we will analyze its effect. In most cases, the increasing order of the partition coefficients of phenolic compounds is in accordance with the log k_ow_ Suc < Ara < Man < Glc < Xyl; however, the exception is vanillin. The recoveries for caffeic and syringic acid in the top phase are less than 50%. However, in the opposite phase of the system, the recoveries of these compounds were higher than 50%. In contrast, values between 50 and 90% recovery at the top for the other phenol compounds demonstrate the possibility of carrying out an elective separation.

**Figure 5:**
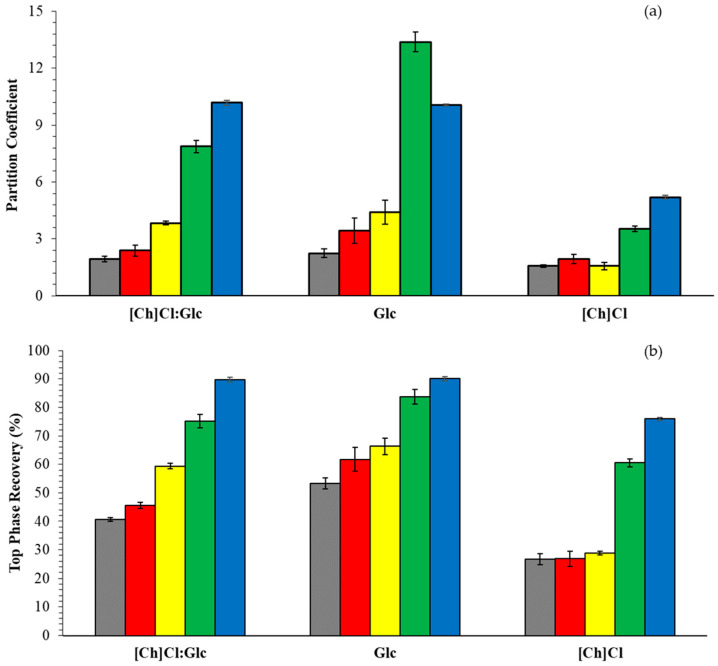
Partition coefficient (**a**) and recovery in top phase (**b**) for ATPS based on ACN + [Ch]Cl:Glc (molar ratio 1:1), Glc, or [Ch]Cl+ water at 25 °C and 0.1 MPa. ■—caffeic acid, ■—syringic acid, ■—vanillic acid, ■—ferulic acid and ■—vanillin.

To compare the different compounds partitioning, Figure 5 (Tables S6 and S7 of Supplementary Materials) depicted the partition coefficient and top phase recovery for the system based on ACN + [Ch]Cl:Glc, Glc, or [Ch]Cl at 25 °C and 0.1 MPa. The systems based on ᴅ-glucose were chosen for the comparison due to the highest K and R_T_ value for DES-based Glu and has a commercial value (USD 60.30/Kg) lower than that of xylose (USD 72.10/Kg) in July 2024.

The system containing d-glucose as a salting-out agent was observed to be the most effective, generating higher partition coefficients of phenolic compounds. The decrease observed with the replacement of Glc by [Ch]Cl may be associated with the higher hydrophilicity of [Ch]Cl. The system based on DES [Ch]Cl:Glc presented intermediate partition coefficient values, possibly influenced by [Ch]Cl. The recoveries in the top phase mostly follow the trend observed for the partition coefficient and varied between 26.71 and 53.36% (caffeic acid), 26.84 and 61.72 (syringic acid), 28.81 and 66.28 (vanillic acid), 60.48 and 83.71 (ferulic acid), and 75.93 and 90.09 (vanillin). It’s well known that DES improves stability, prolongs useful life, in addition to improving antioxidant and anti-inflammatory properties, among others, due to its network of hydrogen bonds formed by natural origin products. Therefore, we believe that using DES to recover biocompounds of pharmaceutical and cosmetic interest is the best way forward. Rosario et al. [66] reported using ACN as an adjuvant to ATPS based on polyethylene glycol (PEG) and potassium phosphate, in which extraction efficiencies of around 80% were achieved for the saline phase for phenolic compounds. Claudio et al. [67] also demonstrated the same partitioning behavior for phenolic compounds in ATPS composed of ionic liquids based on imidazolium and potassium phosphate. Lima et al. [68] achieved extraction values of 98% for anthocyanins from grape skins in systems consisting of [C_2_mim]OAc + potassium phosphate. Meng et al. [69] showed optimum increases in the solubility of phenolic compounds in systems consisting of DES (choline chloride + lactic acid or ethylene glycol) + potassium phosphate.

## 3. Materials and Methods

### 3.1. Materials

The ATPS studied were composed of monosaccharides such as d-glucose (>99 wt%), d-mannose (>98 wt%), d-xylose (>98 wt%), arabinose (>99 wt%), and disaccharides (sucrose, >98 wt%), choline chloride (>98 wt%), and acetonitrile (>99 wt%). The target biomolecules used in the partition studies were caffeic acid (>98 wt%), syringic acid (>98 wt%), vanillic acid (>98 wt%), ferulic acid (>99 wt%), and vanillin (>99 wt%). All the chemical compounds were purchased from Sigma-Aldrich (St. Louis, MO, USA), and ultrapure water was employed in all the experiments (Ultrapure type 1, water Direct-Q3^®^UV, Darmstadt, Germany).

### 3.2. Preparation of Deep Eutectic Solvent

Three molar ratios (1:1, 1:2, and 2:1) of choline chloride (hydrogen-bond acceptor) and carbohydrates (hydrogen-bond donors) were used for preparing the DESs D-(+)glucose:choline chloride ([Ch]Cl:Glc), D(+)-mannose:choline chloride ([Ch]Cl:Man), D-(+)-xylose:choline chloride ([Ch]Cl:Xyl), L-(+)-arabinose:choline chloride ([Ch]ClAra), and sucrose:choline chloride ([Ch]Cl:Suc) by the heating method proposed by Dai et al. [70] with modifications. Briefly, the two-component mixture and sufficient water content for dissolution were added in a round-bottle flask with a stirring bar and closed with a cap. The water content of each DES formed was then measured, and this amount of water was deducted to form the ATPS. The set was heated in a water bath (Marconi MA-127, Piracicapa, Brazil) until the components dissolved and produced a clear liquid (70–100 °C) under vigorous agitation–180 rpm (Tecnal TE-0854, Piracicaba, Brazil).

### 3.3. Binodal Curve

The liquid–liquid equilibrium phase diagrams were built using the cloud-point [71] method and different combinations of constituents (ACN + [CH]Cl + water, ACN + carbohydrates + water, and ACN + DES + water) at 25 °C at 0.10 MPa. Stock solutions of DES (80 wt%) and ACN (80 wt%) were previously prepared and used for the binodal curve determination. The ACN aqueous solution was drop-wise added to the DES aqueous solution under constant magnetic stirring (Tecnal TE-085, Piracicaba, Brazil) until the visual detection of the formation of a cloudy solution (biphasic region), followed by the drop-wise addition of ultrapure water until to observe a clear and limpid solution (monophasic area). The procedure was repeated several times in order to obtain enough points for the binodal curve, which was determined gravimetrically—deviation ± 10^−4^ g (Shimadzu AUW220D, Kyoto, Japan). The binodal curves for the systems composed of ACN + [Ch]Cl + water and ACN + carbohydrates + water at 25 °C and 0.10 MPa were previously reported in the literature [53,54].

### 3.4. Partition of Phenolic Compounds on ATPS

A common mixing point for all the studied ATPS was chosen for the different binodal curves (ACN—30 wt%, DES or carbohydrate, or [Ch]Cl—30 wt% and water 40 wt%) and used for the study of the phenolic compounds partition. The ATPS was prepared in centrifuge tubes (15 mL) by weighing the suitable amount of each constituent of the system and the phenolic compounds (23 mg·L^−1^). The system was vigorously stirred (Tecnal TE-062, Piracicaba, Brazil) and centrifuged at 3000 rpm and 25 °C for 10 min (Hettich Universal 320R, Kirchlengern, Germany). In order to reach the thermodynamic equilibrium, the tubes were left in a thermostatic bath at 25 °C for at least 4 h (Marconi MA-127, Piracicapa, Brazil). The top and bottom phases were carefully separated using a long needle syringe and a pipette, respectively. The volume of each phase was initially determined, and the concentration of phenolic compounds was measured. At least three independent replicates were prepared to determine each parameter’s averages and standard deviations.

The phenolic compounds concentrations were measured in each phase using a spectrophotometer (Varian Cary 50 Bio UV-Vis, Palo Alto, CA, USA) at 317 nm (caffeic acid), 271 nm (syringic acid), 316 nm (ferulic acid), 257 nm (vanillic acid), and 280 nm (vanillin). The calibration curves were previously established using different concentrations of target biomolecules, applying either water (calibration curve) or the corresponding phase being analyzed (partitioning process) as a blank solution, which corresponds to the system without the target biomolecules, i.e., only ACN + DES + water.

The partition coefficient (K) was determined as the ratio between the biomolecule concentration in the top and bottom phases (Equation (1)).

(1) $$K=\frac{C_{T}}{C_{B}}$$

where C represents the phenolic compound concentration and the subscripts T and B represent the top and the bottom phase, respectively.

The phenolic compounds recovery in the top phase was assessed as follows (Equation (2)):

(2) RT=1001+1K × RV

where R_V_ is the volumetric ratio between the volumes of the top and bottom phases.

The selectivity (S) of the systems was determined by the ratio between the partition coefficients of two biomolecules (i and j) under study (Equation (3)).

(3) $$S=\frac{K_{i}}{K_{j}}$$

## 4. Conclusions

Aqueous two-phase system formed by acetonitrile + deep eutectic solvents + water at 25 °C and 0.1 MPa. The DES used in this study was based on choline chloride ([Ch]Cl) and carbohydrates (sucrose, d-glucose, d-mannose, arabinose, and d-xylose). The ability of phase-forming components to form ATPS follows a trend in relation to the number and type of hydroxyls present in the DES-forming carbohydrate, which are the driving forces for phase separation. The higher molar quantity of carbohydrates (1:2) and [Ch]Cl (2:1) in the DES, when compared with the molar ratio 1:1, provides the largest area of phase separation, also resulting from the hydrophilic–hydrophobic balance and possibilities of forming hydrogen bonds with the water in the system. The ATPS were used to study the partition of the phenolic compounds. Due to their hydrophobic compatibility, the phenolic compounds migrated to the top phase (ACN-rich phase). However, one can selectively separate ferulic acid and vanillin for the top phase (R_T_ > 60% and S > 2.05) from caffeic, syringic, and vanillic acids for the bottom phase (R_T_ < 50% and S < 1.96). According to these results, these novel ATPS can be proposed as alternative and economically attractive platforms to recover phenolic compounds from real matrices.

## Data Availability

Data are contained within the article and Supplementary Materials.

## Supplementary Materials

The following supporting information can be downloaded at: https://www.mdpi.com/article/10.3390/molecules29184383/s1, Table S1: Experimental binodal mass fraction data for the system composed of DES 1:1 (1) + ACN (2) + water (3) at 25 °C and 0.1 MPa; Table S2: Experimental binodal mass fraction data for the system composed of DES 1:2 (1) + ACN (2) + water (3) at 25 °C and 0.1 MPa; Table S3: Experimental binodal mass fraction data for the system composed of DES 2:1 (1) + ACN (2) + water (3) at 25 °C and 0.1 MPa; Table S4: Partition coefficients (K) of different biomolecules using ATPS based on DES + ACN + water at 25 °C and 0.10 MPa; Table S5: Top recovery (R_T_) of different biomolecules using ATPS based on DES + ACN + water at 25 °C and 0.10 MPa; Table S6: Partition coefficients (K) of different biomolecules using ATPS based on [Ch]Cl:Glc, Glucose, or [CH]Cl + ACN + water at 25 °C and 0.10 MPa; Table S7: Top recovery (R_T_) of different biomolecules using ATPS based on [Ch]Cl:Glc, Glucose, or [CH]Cl + ACN + water at 25 °C and 0.10 MPa.
