# Supplementary material for: Deep Eutectic Solvent-Based Aqueous Two-Phase Systems and Their Application in Partitioning of Phenol Compounds
Source: Molecules. 2024 Sep 15;29(18):4383. doi: 10.3390/molecules29184383 (PMC11434047; doi:10.3390/molecules29184383)
Supplement: Supplementary file 1 [file molecules-29-04383-s001.zip › molecules-3149321-supplementary.pdf]

Supplementary Materials

Deep Eutectic Solvent-Based Aqueous Two-Phase Systems and Their Application in Partitioning of Phenol Compounds

Isabela N. Souza [1], Lucas C. V. Rodrigues [2], Cleide M. F. Soares [1,3], Filipe S. Buarque [4,*], Ranyere L. Souza [1,3] and Álvaro S. Lima [2,*]

[1] Post-Graduated Program on Process Engineering, Tiradentes University, Aracaju 49032-490, SE, Brazil; isabela__souzza@hotmail.com (I.N.S.); cleide18@yahoo.com.br (C.M.F.S.); ranyerels@hotmail.com (R.L.S.)

[2] Post-Graduate Program on Chemical Engineering, Federal University of Bahia, Salvador 40210-910, BA, Brazil; lucas.rodrigues@yahoo.com.br

[3] Instituto de Tecnologia e Pesquisa, Aracaju 49032-490, SE, Brazil

[4] Biochemical Engineering Department, School of Chemistry, Federal University of Rio de Janeiro, Rio de Janeiro 21941-909, RJ, Brazil

* Correspondence: filipebuarque@ua.pt (F.S.B.); aslima2001@yahoo.com.br (Á.S.L.)

Table S1. Experimental binodal mass fraction data for the system composed of DES 1:1 (1) + ACN (2) + water (3) at 25ºC and 0.1 MPa.[a]

[Ch]Cl:Man (1:1)			[Ch]Cl:Ara (1:1)			[Ch]Cl:Suc (1:1)			[Ch]Cl:Xyl (1:1)			[Ch]Cl:Glc (1:1)		
[Ch]Cl:Man ($100w_1$)	ACN ($100w_2$)	H_2O ($100w_3$)	[Ch]Cl:Ara ($100w_1$)	ACN ($100w_2$)	H_2O ($100w_3$)	[Ch]Cl:Suc ($100w_1$)	ACN ($100w_2$)	H_2O ($100w_3$)	[Ch]Cl:Xyl ($100w_1$)	ACN ($100w_2$)	H_2O ($100w_3$)	[Ch]Cl:Glc ($100w_1$)	ACN ($100w_2$)	H_2O ($100w_3$)
70.05	12.63	17.31	70.11	12.63	17.26	64.90	17.37	17.73	70.18	12.61	17.21	65.66	16.68	17.66
60.25	13.20	26.55	59.59	13.22	27.20	50.54	17.20	32.26	54.32	16.79	28.89	54.21	16.00	29.79
48.55	14.49	36.96	47.55	15.99	36.47	36.96	18.74	44.30	45.13	19.93	34.94	40.67	16.65	42.69
43.44	16.76	39.81	40.56	17.77	41.67	32.38	20.52	47.10	36.85	22.19	40.96	37.71	17.88	44.41
38.76	18.20	43.05	36.30	19.42	44.28	29.13	22.19	48.67	31.41	24.40	44.19	35.99	18.47	45.54
34.74	19.92	45.35	32.30	21.18	46.52	26.33	23.80	49.87	27.90	25.29	46.81	34.32	19.23	46.45
32.54	20.32	47.14	28.39	22.96	48.64	24.15	25.54	50.31	25.62	26.47	47.91	32.97	19.67	47.37
29.86	21.94	48.20	25.21	25.63	49.16	22.22	26.75	51.03	23.57	27.63	48.80	31.82	20.93	47.25
27.61	23.40	48.98	22.43	26.76	50.81	20.72	28.17	51.11	21.93	29.70	48.37	30.42	21.60	47.98
25.71	24.50	49.79	20.48	28.23	51.29	19.41	29.39	51.20	20.45	30.96	48.59	29.09	22.21	48.70
23.89	25.72	50.39	18.97	29.36	51.67	18.16	30.67	51.17	19.11	32.10	48.80	27.95	22.83	49.22
22.09	27.08	50.82	17.77	31.13	51.09	17.12	31.64	51.24	18.04	33.43	48.53	27.04	23.51	49.45
20.46	28.67	50.87	16.52	32.22	51.26	16.07	32.79	51.14	16.64	35.20	48.16	26.06	24.06	49.88
18.81	29.90	51.29	15.37	33.83	50.80	15.22	34.17	50.61	15.56	36.86	47.58	25.09	24.80	50.11
17.73	31.45	50.82	14.40	35.07	50.53	14.46	35.34	50.20	14.46	38.36	47.17	24.59	25.26	50.15
16.40	32.52	51.07	13.27	36.95	49.77	13.73	36.52	49.75	13.52	39.85	46.62	23.94	25.93	50.13
15.59	33.71	50.70	12.42	38.24	49.34	13.09	37.72	49.20	12.71	41.37	45.92	23.20	26.34	50.46
14.69	34.87	50.44	11.30	40.76	47.94	12.60	38.81	48.59	11.75	43.50	44.75	22.53	26.75	50.72
13.85	36.51	49.65	10.46	42.41	47.13	12.04	39.83	48.14	11.05	45.33	43.63	22.11	27.13	50.76
12.99	37.65	49.35	9.94	43.88	46.18	11.53	41.05	47.42				21.77	27.51	50.72
11.98	38.64	49.38				11.06	42.20	46.73				21.45	27.62	50.93
11.27	40.21	48.52				10.48	43.49	46.03				20.77	28.39	50.84
10.74	41.29	47.97				10.10	44.43	45.47				20.22	29.09	50.69

[a] Standard uncertainties u are u([DES]. [ACN] or [H_2O]) = 0.01. u(T) = 0.2ºC, and u(p) = 10 kPa.

Table S1 cont. Experimental binodal mass fraction data for the system composed of DES 1:1 (1) + ACN (2) + water (3) at 25ºC and 0.1 MPa.[a]

[Ch]Cl:Man (1:1)			[Ch]Cl:Ara (1:1)			[Ch]Cl:Suc (1:1)			[Ch]Cl:Xyl (1:1)			[Ch]Cl:Glc (1:1)		
[Ch]Cl:Man ($100w_1$)	ACN ($100w_2$)	H_2O ($100w_3$)	[Ch]Cl:Ara ($100w_1$)	ACN ($100w_2$)	H_2O ($100w_3$)	[Ch]Cl:Suc ($100w_1$)	ACN ($100w_2$)	H_2O ($100w_3$)	[Ch]Cl:Xyl ($100w_1$)	ACN ($100w_2$)	H_2O ($100w_3$)	[Ch]Cl:Glc ($100w_1$)	ACN ($100w_2$)	H_2O ($100w_3$)
10.28	42.56	47.16				9.71	45.42	44.87				19.38	30.02	50.61
9.79	44.01	46.20										18.58	30.93	50.49
9.23	45.67	45.10										17.90	31.45	50.65
8.66	47.47	43.87										17.51	31.93	50.56
7.41	51.64	40.94										17.00	32.43	50.57
												16.39	33.14	50.47
												16.03	33.72	50.26
												15.43	34.54	50.03
												15.04	35.27	49.69
												14.51	36.02	49.48
												13.97	36.78	49.25
												13.61	37.50	48.88
												13.28	38.09	48.62
												12.92	38.59	48.49
												12.60	39.23	48.17
												12.32	39.79	47.89
												12.07	40.39	47.54
												11.76	41.15	47.09
												11.46	41.78	46.76
												11.05	42.83	46.12
												10.63	43.96	45.41
												10.41	44.38	45.21
												10.14	45.03	44.83

[a] Standard uncertainties u are u([DES]. [ACN] or [H_2O]) = 0.01. u(T) = 0.2ºC, and u(p) = 10 kPa.

Table S1 cont. Experimental binodal mass fraction data for the system composed of DES 1:1 (1) + ACN (2) + water (3) at 25ºC and 0.1 MPa.[a]

[Ch]Cl:Man (1:1)			[Ch]Cl:Ara (1:1)			[Ch]Cl:Suc (1:1)			[Ch]Cl:Xyl (1:1)			[Ch]Cl:Glc (1:1)		
[Ch]Cl:Man ($100w_1$)	ACN ($100w_2$)	H_2O ($100w_3$)	[Ch]Cl:Ara ($100w_1$)	ACN ($100w_2$)	H_2O ($100w_3$)	[Ch]Cl:Suc ($100w_1$)	ACN ($100w_2$)	H_2O ($100w_3$)	[Ch]Cl:Xyl ($100w_1$)	ACN ($100w_2$)	H_2O ($100w_3$)	[Ch]Cl:Glc ($100w_1$)	ACN ($100w_2$)	H_2O ($100w_3$)
												9.82	45.91	44.28
												9.45	46.96	43.60

[a] Standard uncertainties u are u([DES]. [ACN] or [H_2O]) = 0.01. u(T) = 0.2ºC, and u(p) = 10 kPa.

Table S2. Experimental binodal mass fraction data for the system composed of DES 1:2 (1) + ACN (2) + water (3) at 25ºC and 0.1 MPa.[a]

[Ch]Cl:Man (1:2)			[Ch]Cl:Ara (1:2)			[Ch]Cl:Suc (1:2)			[Ch]Cl:Xyl (1:2)			[Ch]Cl:Glc (1:2)		
[Ch]Cl:Man ($100w_1$)	ACN ($100w_2$)	H_2O ($100w_3$)	[Ch]Cl:Ara ($100w_1$)	ACN ($100w_2$)	H_2O ($100w_3$)	[Ch]Cl:Suc ($100w_1$)	ACN ($100w_2$)	H_2O ($100w_3$)	[Ch]Cl:Xyl ($100w_1$)	ACN ($100w_2$)	H_2O ($100w_3$)	[Ch]Cl:Glc ($100w_1$)	ACN ($100w_2$)	H_2O ($100w_3$)
71.94	10.89	17.18	71.50	11.18	17.32	68.99	13.66	17.36	68.05	14.65	17.30	64.62	17.72	17.65
60.16	12.06	27.78	59.87	12.46	27.67	54.77	14.82	30.41	47.59	19.28	33.13	50.90	17.08	32.02
47.80	13.95	38.26	47.97	14.73	37.31	41.10	16.71	42.18	35.75	21.34	42.91	36.61	19.15	44.24
42.50	16.27	41.23	43.32	16.70	39.98	36.13	18.68	45.19	30.30	23.40	46.30	31.52	20.97	47.51
37.76	18.03	44.21	39.37	18.59	42.04	31.76	20.64	47.60	26.09	25.26	48.65	27.84	22.97	49.19
33.71	19.56	46.74	35.63	19.72	44.64	28.80	21.90	49.30	23.24	27.10	49.66	24.77	24.28	50.96
30.16	21.19	48.65	32.89	20.99	46.12	26.29	23.56	50.15	20.80	28.38	50.82	22.46	26.11	51.42
27.86	22.68	49.47	29.70	22.83	47.47	24.25	24.78	50.96	19.15	30.22	50.63	20.59	27.52	51.88
25.17	24.40	50.43	27.28	23.99	48.73	22.45	26.04	51.51	17.82	31.56	50.62	19.07	28.54	52.39
22.86	26.27	50.87	25.17	25.40	49.43	21.02	27.48	51.51	16.59	33.06	50.35	17.70	30.10	52.20
20.75	27.47	51.77	23.20	26.58	50.22	19.54	28.53	51.93	15.69	34.51	49.80	16.63	31.31	52.06
19.01	29.30	51.70	21.66	28.15	50.19	18.56	29.82	51.62	13.83	38.35	47.83	15.74	32.56	51.70
17.56	31.37	51.07	19.82	29.80	50.38	17.48	31.08	51.44	12.47	40.58	46.95	14.78	33.85	51.37
16.38	31.81	51.81	18.71	30.78	50.51	16.46	32.07	51.48	11.24	44.01	44.75	14.05	35.07	50.88
15.55	33.11	51.34	17.57	32.02	50.42	15.64	33.48	50.88	10.28	46.29	43.43	13.26	36.04	50.69
14.71	34.42	50.87	16.56	33.03	50.41	14.82	34.57	50.61	9.73	47.76	42.51	12.73	37.29	49.98
13.50	36.58	49.92	15.61	34.13	50.25	15.61	14.14	35.70	50.16	9.29	48.87	12.07	38.22	49.71
12.58	37.55	49.87	14.81	35.50	49.70	14.81	13.50	36.80	49.70	8.74	50.35	11.49	39.38	49.13
11.89	38.93	49.19	14.11	36.60	49.29	14.11	12.94	37.80	49.26			10.92	40.82	48.26
11.37	39.87	48.76	13.34	38.03	48.63	13.34	12.38	38.83	48.79			10.45	41.80	47.76
10.66	41.55	47.79	12.70	39.27	48.03	12.70	11.95	39.81	48.24			9.93	42.93	47.13
9.92	43.42	46.66	12.00	40.73	47.26	12.00	11.34	41.02	47.64			9.51	44.13	46.36
9.18	45.48	45.33	11.25	42.42	46.33	11.25	10.77	42.32	46.91			9.02	45.61	45.38

[a] Standard uncertainties u are u([DES]. [ACN] or [H_2O]) = 0.01. u(T) = 0.2ºC, and u(p) = 10 kPa.

Table S2 cont. Experimental binodal mass fraction data for the system composed of DES 1:2 (1) + ACN (2) + water (3) at 25ºC and 0.1 MPa.[a]

[Ch]Cl:Man (1:2)			[Ch]Cl:Ara (1:2)			[Ch]Cl:Suc (1:2)			[Ch]Cl:Xyl (1:2)			[Ch]Cl:Glc (1:2)		
[Ch]Cl:Man ($100w_1$)	ACN ($100w_2$)	H_2O ($100w_3$)	[Ch]Cl:Ara ($100w_1$)	ACN ($100w_2$)	H_2O ($100w_3$)	[Ch]Cl:Suc ($100w_1$)	ACN ($100w_2$)	H_2O ($100w_3$)	[Ch]Cl:Xyl ($100w_1$)	ACN ($100w_2$)	H_2O ($100w_3$)	[Ch]Cl:Glc ($100w_1$)	ACN ($100w_2$)	H_2O ($100w_3$)
8.74	46.82	44.43	10.82	43.49	45.68	10.82	10.35	43.48	46.17			8.59	46.91	44.51
8.31	48.15	43.54	10.35	44.76	44.89	10.35	10.01	44.39	45.60			8.16	48.22	43.62
			9.74	46.42	43.84	9.74	9.54	45.42	45.04					

[a] Standard uncertainties u are u([DES]. [ACN] or [H_2O]) = 0.01. u(T) = 0.2ºC, and u(p) = 10 kPa.

Table S3. Experimental binodal mass fraction data for the system composed of DES 2:1 (1) + ACN (2) + water (3) at 25°C and 0.1 MPa.[a]

[Ch]Cl:Man (2:1)			[Ch]Cl:Ara (2:1)			[Ch]Cl:Suc (2:1)			[Ch]Cl:Xyl (2:1)			[Ch]Cl:Glc (2:1)		
[Ch]Cl:Man ($100w_1$)	ACN ($100w_2$)	H_2O ($100w_3$)	[Ch]Cl:Ara ($100w_1$)	ACN ($100w_2$)	H_2O ($100w_3$)	[Ch]Cl:Suc ($100w_1$)	ACN ($100w_2$)	H_2O ($100w_3$)	[Ch]Cl:Xyl ($100w_1$)	ACN ($100w_2$)	H_2O ($100w_3$)	[Ch]Cl:Glc ($100w_1$)	ACN ($100w_2$)	H_2O ($100w_3$)
69.73	13.00	17.27	71.69	10.98	17.33	60.06	22.26	17.68	64.06	18.43	17.51	69.29	13.40	17.31
59.86	14.01	26.13	61.56	12.90	25.54	45.51	21.38	33.11	47.61	20.59	31.80	50.58	18.12	31.30
48.99	15.61	35.40	53.79	14.12	32.09	32.58	22.92	44.49	36.53	22.54	40.94	41.54	20.26	38.20
44.44	17.08	38.48	48.22	16.06	35.73	28.62	24.72	46.66	30.90	24.17	44.93	35.06	21.52	43.42
40.00	18.70	41.30	42.91	17.70	39.39	25.87	26.26	47.86	27.19	26.03	46.78	31.28	23.13	45.59
36.44	20.61	42.94	38.61	19.18	42.21	23.51	27.80	48.69	24.54	27.67	47.79	28.04	24.58	47.38
33.55	21.70	44.75	35.18	20.17	44.65	21.43	29.09	49.48	22.24	29.49	48.26	25.89	25.86	48.25
30.33	23.04	46.63	32.43	21.60	45.98	19.92	30.27	49.81	20.37	30.96	48.67	24.10	27.02	48.88
27.85	24.69	47.46	29.85	23.13	47.01	18.79	31.66	49.55	18.72	32.04	49.24	22.59	28.12	49.29
26.15	25.35	48.50	27.99	23.76	48.25	17.52	32.88	49.60	17.44	33.37	49.18	21.10	29.18	49.71
24.73	26.33	48.94	25.71	25.02	49.27	16.58	34.28	49.14	16.22	34.99	48.79	19.67	30.37	49.96
22.86	27.80	49.34	23.57	26.64	49.78	15.58	35.85	48.57	15.24	36.61	48.15	18.61	31.34	50.05
21.06	29.41	49.53	22.39	27.25	50.36	14.72	37.27	48.01	14.29	38.01	47.70	17.77	32.43	49.80
19.32	30.71	49.96	20.61	28.60	50.79	13.88	38.64	47.48	13.51	39.32	47.17	16.91	33.03	50.06
18.14	31.69	50.17	19.51	29.97	50.53	12.88	40.01	47.11	12.67	40.88	46.45	16.19	33.84	49.97
17.05	33.25	49.70	18.27	31.21	50.52	12.14	41.35	46.51	12.07	42.36	45.58	15.56	34.58	49.86
15.87	34.60	49.54	17.21	32.30	50.49	11.45	42.80	45.75	11.43	43.64	44.93	15.05	35.45	49.50
14.81	35.80	49.39	16.20	33.49	50.32	10.90	44.17	44.94	10.77	45.30	43.93	14.50	36.26	49.24
14.09	36.88	49.03	15.31	34.72	49.97	10.28	45.41	44.31	10.13	47.01	42.86	13.94	37.20	48.86
13.33	38.14	48.53	14.38	35.95	49.67	9.75	46.83	43.43				13.46	38.07	48.47
12.64	39.25	48.11	13.75	36.98	49.27	9.19	48.33	42.48				12.99	38.85	48.16
11.87	40.74	47.40	12.83	38.68	48.49	8.60	50.03	41.37				12.50	39.81	47.69
11.00	42.67	46.33	12.19	39.87	47.94	8.17	51.23	40.59				12.07	40.75	47.18

[a] Standard uncertainties u are u([DES]. [ACN] or [H_2O]) = 0.01. u(T) = 0.2°C, and u(p) = 10 kPa.

Table S3 cont. Experimental binodal mass fraction data for the system composed of DES 2:1 (1) + ACN (2) + water (3) at 25ºC and 0.1 MPa.[a]

[Ch]Cl:Man (2:1)			[Ch]Cl:Ara (2:1)			[Ch]Cl:Suc (2:1)			[Ch]Cl:Xyl (2:1)			[Ch]Cl:Glc (2:1)		
[Ch]Cl:Man ($100w_1$)	ACN ($100w_2$)	H_2O ($100w_3$)	[Ch]Cl:Ara ($100w_1$)	ACN ($100w_2$)	H_2O ($100w_3$)	[Ch]Cl:Suc ($100w_1$)	ACN ($100w_2$)	H_2O ($100w_3$)	[Ch]Cl:Xyl ($100w_1$)	ACN ($100w_2$)	H_2O ($100w_3$)	[Ch]Cl:Glc ($100w_1$)	ACN ($100w_2$)	H_2O ($100w_3$)
10.23	44.11	45.67	11.09	42.36	46.55	7.78	52.06	40.16				11.66	41.64	46.70
9.62	45.87	44.51	10.42	43.96	45.62							11.51	41.92	46.57
9.10	47.35	43.55	9.94	45.28	44.78							11.04	42.13	46.82
8.50	49.16	42.34										10.78	42.83	46.39
												10.45	43.46	46.09
												10.05	44.59	45.35
												9.53	46.19	44.28
												9.28	46.82	43.91

[a] Standard uncertainties u are u([DES]. [ACN] or [H_2O]) = 0.01. u(T) = 0.2ºC,and u(p) = 10 kPa.

Table S4. Partition coefficients (K) of different biomolecules using ATPS based on DES + ACN + water at 25°C and 0.10 MPa[a].

DES	K_{SYRA}	K_{VANA}	K_{CAFA}	K_{FERA}	K_{VANI}
[Ch]Cl:Man	2.20 ± 0.03	2.77 ± 0.10	1.65 ± 0.08	4.98 ± 0.31	5.25 ± 0.10
[Ch]Cl:Glc	2.39 ± 0.29	3.83 ± 0.09	1.95 ± 0.15	7.87 ± 0.32	10.18 ± 0.13
[Ch]Cl:Xyl	2.71 ± 0.59	3.88 ± 0.16	2.05 ± 0.52	7.89 ± 0.54	8.09 ± 0.25
[Ch]Cl:Ara	2.19 ± 0.21	3.08 ± 0.16	1.55 ± 0.15	6.30 ± 0.87	7.14 ± 0.47
[Ch]Cl:Suc	1.87 ± 0.52	2.58 ± 0.15	1.42 ± 0.12	4.47 ± 0.22	5.71 ± 0.17

[a] Standard uncertainties u are u(T) = 0.2°C, and u(p) = 10 kPa.

Table S5. Top recovery (R_T) of different biomolecules using ATPS based on DES + ACN + water at at 25°C and 0.10 MPa[a].

DES	$(R_T)_{SYRA}$	$(R_T)_{VANA}$	$(R_T)_{CAFA}$	$(R_T)_{FERA}$	$(R_T)_{VANI}$
[Ch]Cl:Man	42.13 ± 1.94	49.59 ± 0.18	39.09 ± 0.21	62.96 ± 1.16	70.58 ± 2.35
[Ch]Cl:Glc	45.56 ± 0.99	59.32 ± 0.94	40.57 ± 0.67	75.20 ± 2.39	89.75 ± 0.78
[Ch]Cl:Xyl	43.75 ± 4.02	52.99 ± 1.74	37.83 ± 4.93	70.93 ± 3.09	75.89 ± 3.89
[Ch]Cl:Ara	40.66 ± 1.02	52.68 ± 7.31	34.85 ± 2.47	60.06 ± 2.58	63.45 ± 4.89
[Ch]Cl:Suc	39.23 ± 6.16	48.11 ± 2.26	37.75 ± 0.80	67.98 ± 0.96	69.99 ± 3.81

[a] Standard uncertainties u are u(T) = 0.2°C, and u(p) = 10 kPa.

Table S6. Partition coefficients (K) of different biomolecules using ATPS based on [Ch]Cl:Glc, Glucose or [CH]Cl + ACN + water at 25°C and 0.10 MPa[a].

DES	K_{SYRA}	K_{VANA}	K_{CAFA}	K_{FERA}	K_{VANI}
[Ch]Cl:Glc	2.39 ± 0.29	3.83 ± 0.09	1.95 ± 0.15	7.87 ± 0.32	10.18 ± 0.13
Glc	3.44 ± 0.68	4.40 ± 0.63	2.24 ± 0.23	13.39 ± 0.51	10.06 ± 0.05
[CH]Cl	1.93 ± 0.24	1.58 ± 0.20	1.57 ± 0.05	3.52 ± 0.15	5.20 ± 0.11

[a] Standard uncertainties u are u(T) = 0.2°C, and u(p) = 10 kPa.

Table S7. Top recovery (R_T) of different biomolecules using ATPS based on [Ch]Cl:Glc, Glucose or [CH]Cl + ACN + water at 25°C and 0.10 MPa[a].

DES	$(R_T)_{SYRA}$	$(R_T)_{VANA}$	$(R_T)_{CAFA}$	$(R_T)_{FERA}$	$(R_T)_{VANI}$
[Ch]Cl:Gl	45.56 ± 0.99	59.32 ± 0.94	40.57 ± 0.67	75.20 ± 2.39	89.75 ± 0.78
Glc	61.72 ± 4.11	66.28 ± 2.91	53.36 ± 1.93	83.71 ± 2.62	90.09 ± 0.78
[CH]Cl	26.84 ± 2.67	28.81 ± 0.64	26.71 ± 1.93	60.48 ± 1.46	75.93 ± 0.55

[a] Standard uncertainties u are u(T) = 0.2°C, and u(p) = 10 kPa.
